# The prognostic utility of IGF-1 in hepatocellular carcinoma treated with stereotactic body radiotherapy

**DOI:** 10.1016/j.ctro.2024.100887

**Published:** 2024-11-12

**Authors:** Ahmed Allam Mohamed, Cennet Sahin, Marie-Luise Berres, Oliver Beetz, Martin von Websky, Thomas Vogel, Florian W.R. Vondran, Philipp Bruners, Matthias Imöhl, Katharina Frank, Edith Vogt, Binney Pal Singh, Michael J. Eble

**Affiliations:** a Radiation Oncology Department, University Hospital RWTH Aachen, Aachen, Germany; b Gastroenterology, Hepatology and Infectious Diseases Department, University Hospital RWTH Aachen, Aachen, Germany; c General, Visceral, Pediatric and Transplantation Surgery Department, University Hospital RWTH Aachen, Aachen, Germany; d Diagnostic and Interventional Radiology Department, University Hospital RWTH Aachen, Aachen, Germany; e Laboratory Diagnostic Center, University Hospital RWTH Aachen, Aachen, Germany; f Center for Integrated Oncology Aachen, Bonn, Cologne and Duesseldorf (CIO ABCD), Aachen, Germany

**Keywords:** Hepatocellular carcinoma, IGF-1, SBRT, Survival analysis, Nomogram, Liver cancer prognosis

## Abstract

**Background:**

Hepatocellular carcinoma (HCC) poses a significant challenge for patients ineligible for surgical resection or liver transplantation. Local therapies like Stereotactic Body Radiotherapy (SBRT) are crucial for those with liver-limited disease. Insulin-like growth factor-1 (IGF-1) is a potential biomarker for liver function. This study evaluates IGF-1’s prognostic value in predicting survival outcomes in HCC patients undergoing SBRT.

**Methods:**

We analyzed 42 HCC patients treated with SBRT between May 2021 and January 2024, with IGF-1 levels measured within four weeks before SBRT. Patient demographics, tumor metrics, and clinical outcomes were examined. The prognostic significance of IGF-1 was assessed using Cox proportional hazards and ROC curve analysis to determine optimal IGF-1 cutoffs for survival prediction. A nomogram predicting 1-year and 2-year survival was constructed using a multivariate Cox model.

**Results:**

IGF-1 levels were significantly lower in patients with cirrhosis or sarcopenia. Median overall survival (OS) was 24 months, with a significant survival difference favoring patients with IGF-1 levels above 62.4 ng/ml (Hazard Ratio [HR]: 5.9, P = 0.0025). A multivariable Cox model including Child-Turcotte-Pugh (CTP) score, IGF-1, and tumor volume effectively predicted survival. IGF-1 and tumor volume significantly impacted OS (HR: 6.9 and 1.004, p = 0.014 and 0.0022, respectively). Integrating IGF-1 with CTP score improved predictive accuracy (c-index 0.66 to 0.75, p = 0.052).

The nomogram, integrating IGF-1 with the CTP and tumour volume, exhibited robust predictive accuracy with an area under the curve (AUC) of 0.84 for 2-year survival.

**Conclusion:**

IGF-1 is a reliable biomarker for liver function and survival prediction in HCC patients undergoing SBRT. Higher IGF-1 levels indicate better prognosis. The developed nomogram, incorporating IGF-1, enhances clinical decision-making for SBRT management. Further validation in larger cohorts is needed.

## Highlights

• IGF-1 mirrors liver's synthetic capacity, reflecting functional reserve and serving as a biomarker of liver health.
• Cirrhosis and sarcopenia lower serum IGF-1, highlighting their combined impact on liver function and reserve.
• Reduced IGF-1 links to worse OS but not PFS, marking its role in predicting liver reserve post-SBRT.
• Combining IGF-1 with CTP score improves precision in functional liver reserve assessment.
• A nomogram using IGF-1, CTP score, and tumor volume predicts 2-year survival in HCC patients post-SBRT.

## Introduction

Primary liver cancer is the sixth most prevalent cancer worldwide, with approximately 830,180 deaths annually, accounting for the third leading cause of cancer-related mortality, with hepatocellular carcinoma (HCC) constituting the majority of primary liver cancers [1], [2]. While surgical resection and orthotopic liver transplantation are traditionally considered definitive treatments, they are limited by strict eligibility criteria [3], [4]. In recent years, Stereotactic Body Radiotherapy (SBRT) has significantly advanced in treating HCC, showcasing remarkable efficacy across diverse disease stages, and acting as a potent bridging strategy prior to liver transplantation [5], [6], [7], [8], [9]. Numerous studies consistently affirm that SBRT delivers exceptional local control rates beyond 90 % and outperforms other locoregional therapies [10], [11], [12], [13]. Despite these impressive results, a major challenge remains that patients' survival is often limited despite the high control rates achieved [8]. This disparity between superior tumour control and restricted patient longevity is typically attributed to the rapid deterioration of liver function and subsequent hepatic cell failure [14].

Insulin-like growth factors (IGFs) are polypeptide hormones that are dependent on growth hormone (GH) and stimulate cell replication across most mesenchymal-derived tissues, underpinning the growth-promoting effects of GH [15]. There are two types of IGFs: IGF-1 and IGF-2. IGF-1 is primarily synthesised in response to GH and is regulated by hypothalamic signals including GH-releasing hormone and somatostatin, as well as by feedback from IGF-1 itself and ghrelin, a gastric hormone [16]. Approximately 75 % of the liver-produced IGF-1 circulates to mediate systemic endocrine functions. In contrast, the remaining 25 % is synthesised in tissues such as bones, cartilage, the central nervous system, kidneys, ovaries, and erythroid cell precursors and exerts localised effects through autocrine and paracrine mechanisms [16].

As liver function deteriorates, the ability of the liver to produce IGF-1 diminishes, establishing IGF-1 as a crucial surrogate marker of liver health [17], [18], [19]. Clinically, reduced levels of IGF-1 are frequently associated with advanced stages of liver disease such as cirrhosis and HCC [19], [20]. This association is particularly valuable for gauging the severity of liver conditions, offering clearer insights where traditional diagnostic methods may fall short due to the complex influences of factors like inflammation and fibrosis [16], [20]. In addition, unlike other liver function tests, IGF-1 levels remain relatively unaffected by acute phase reactions, thus providing a more consistent indicator of the long-term liver reserve. This consistency renders IGF-1 an effective tool for monitoring disease progression or gauging recovery following various treatments, including surgical resection, liver transplantation, or locoregional therapies [21], [22], [23], [24].

The prognostic utility of IGF-1 in the context of treating HCC with SBRT has not been fully investigated. In this study, we aimed to investigate the prognostic accuracy of IGF-1 levels, assess their implications for this particular treatment modality, and construct a nomogram to predict survival.

## Material and methods

This analysis was conducted on patients with HCC who received liver-directed SBRT as part of their management in “RWTH Aachen University Hospital” and had the serum IGF-1 level measured within four weeks before SBRT started. The measurement of IGF-1 was performed using the LIAISON® XL analyser, a fully automated chemiluminescence immunoassay system (DiaSorin S.p.A.) using the LIAISON® IGF-I assay kit for the quantification of IGF-1 levels in serum samples as previously described [25]. Patients without prior measurement of IGF-1 or who received SBRT other than to the liver were excluded from the analysis. The indication and delivery of SBRT have been described [5].

Clinical data examined included age, sex, body mass index, cirrhosis, laboratory liver function tests, liver volume, alpha-fetoprotein (AFP), tumour size and volume, macroscopic vascular invasion, and distant metastases.

The Child-Turcotte-Pugh (CTP) score was estimated following the established method [26]. Similarly, the IGF1-CTP score for each patient was derived as previously outlined [20], [22]. Specifically, IGF1-CTP scores incorporate points assigned based on levels of albumin, bilirubin, INR, and IGF-1, where IGF-1 scoring was performed as follows: more than 50 ng/ml gives 1 point; 26–50 ng/ml gives 2 points; and less than 26 ng/ml accrues 3 points. Aggregate scores ranging from 4 to 5 categorise patients into IGF1-CTP Class A, scores of 6 to 7 in Class B, and scores exceeding 7 in Class C.

The study was approved by the local ethics committee (Faculty of Medicine, RWTH Aachen University, EK 23-264). The analysis and model reporting were guided by the principles of the TRIPOD statement, and relevant items from the checklist (Appendix 1) were considered [27].

### Sarcopenia measurement

Before initiating SBRT, all patients underwent contrast-enhanced multislice planning computed tomography (P-CT) using a 16-slice CT scanner (Brilliance CT Big Bore Oncology, Philips Medical Systems Inc., Cleveland, OH, USA). The scans were conducted at 120 kVp with a slice thickness of 2 mm and a reconstructed pixel size of 1.17 mm × 1.17 mm. Subsequently, the P-CT images were exported in DICOM format to the 3D Slicer segmentation software for further analysis [28]. Measurements were specifically performed on a single image at the mid-lumbar vertebrae L3 level. CT attenuation thresholds ranging from −29 to 150 Hounsfield Units (HU) were applied for the semi-automated skeletal muscle surface area (SMA) delineation. The skeletal muscle index (SMI) was calculated using the formula.

SMA/height^2^

Sarcopenia was diagnosed based on SMI thresholds of less than 41 cm^2^/m^2^ for females and less than 53 cm^2^/m^2^ for males [29].

### Statistical analysis

The primary endpoint of the analysis was overall survival (OS), which is defined as the interval from the initiation of SBRT to the time of death or censoring. Progression-free survival (PFS) was defined as the interval from initiating the radiation treatment to the point of any site disease progression or censoring. Freedom from local progression (FFLP) is defined at the treated lesion level as the time from radiation initiation until the subsequent local progression or censored. In the case of a liver transplant, FFLP, PFS, and OS for the respective patient were censored at the time of transplant.

Pearson correlation analysis was used to examine the relationship between IGF-1 levels and patients’ characteristics. The Mann-Whitney *U* test was used to compare the medians of non-parametric data. Receiver operating characteristic (ROC) curve analysis was conducted to determine the most statistically robust cut-point for significance. Kaplan-Meier analysis was applied to estimate the survival parameters, and univariate and multivariate survival analyses were performed using the Cox proportional hazards model, from which hazard ratios (HRs) and 95 % confidence intervals (CIs) were derived. Additionally, concordance (c) statistics were applied to evaluate the model's predictive accuracy in estimating overall survival. The time-dependent ROC analysis with the area under the curve (AUC) was applied to compare the predictive accuracy of different models using the “timeROC” package. For establishing and validating the nomogram, the “rms” package was used and 1000 bootstrap resamples were generated and used to validate the nomogram. The statistical analysis and graphics were performed using R software version 4.3.1.

## Results

Between May 2021 and January 2024, 43 patients underwent liver-directed SBRT for HCC after being deemed unresectable or as bridging therapy before liver transplantation. IGF-1 levels were prospectively measured before SBRT in 42 patients, and only one patient was excluded from the analysis due to the lack of IGF-1 measurement before radiation treatment. Survival and disease progression data were available for all 42 patients and 39 patients with 52 treated lesions, respectively. Patients’ characteristics are detailed in Table 1. Sixteen patients received SBRT as their first therapy, while 26 were previously treated, as detailed in Table 1.

**Table 1:** Patients’ characteristics. IGF-1: IGF-1 Insulin-like growth factor 1, ALT: Alanine transaminase, AST: Aspartate transaminase, GGT: Gamma-glutamyltransferase, INR: international normalized ratio, CTP: Child-Turcotte-Pugh, (BCLC) Barcelona Clinic Liver Cancer staging system, TACE: transarterial chemoembolization, SIRT: selective internal radiation therapy, AFP: Alpha-Fetoprotein, BMI: body mass index, EQD2*:* median equivalent dose in 2  Gy per fraction, Gy Gray.

Characteristic	
Age	74 (50–87)
Gender - Female - Male	16 26
Cirrhosis: No cirrhosis: Cirrhosis	7 (16.7 %)35 (83.3 %)
Median IGF-1 (range)	62.4 (21.3–161.4) ng/ml
Median Albumin (range)	3.75 (2.5–4.7) g/dl
Median Total Bilirubin (range)	0.81 (0.21–3.38) mg/dl
Median ALT (range)	29 (10–211) U/L
Median AST (range)	43 (18–132) U/L
Median GGT (range)	175 (14–909) U/L
Median INR	1.16 (0.97–2.44)
Ascites: - No - Mild - Moderate to sever	31 3 8
CTP score - A - B - C	27 12 3
IGF1-CTP score: - A - B - C	26 12 4
Change in CTP score 3 months post SBRT. unchanged - 1 point increase - 2 points increase. - I point decrease (improvement)	28 4 1 2
Tumor size: - <2 cm - 2–5 cm - >5	8 16 18
BCLC Stage: A B C D	15 18 6 3
Prior Therapy: No Yes: Surgical resection SIRT Systemic therapy Thermal ablation TACE	16 26 9 1 4 1 14
Therapy after SBRT No Yes: Systemic therapy Second course SBRT Thermal ablation Liver Transplant	23 19 10 3 4 4
Median Liver volume (range)	1407 (657–2545) cm^3^
Median Tumor volume (range)	20.3 (0.7–981) cm^3^
Median AFP (range)	11.5 (2.4–9066) ng/ml
Sarcopenia - Yes - No	19 23
Median BMI (range)	25.7 (17.58–43.95) kg/m^2^
Median Physical prescribed dose Median number of fractionsMedina EQD2 (range)	40 (18–50) Gy5 (1–8) Fractions60 (31.25–83.3) Gy

Four patients underwent liver transplantation at 1.5, 2.4, 6.2, and 10 months post-SBRT; the survival and disease progression data for these patients were censored at the time of transplantation. With a median follow-up of 15.4 months (interquartile range [IQR]: 8.5–24.4 months), the median OS was 24 months. OS rates at one and three years were 59.4 % (95 % CI: 45–79 %) and 46 % (95 % CI: 28–74 %), respectively. PFS was 18.2 months. FFLP rates were 92.5 % and 81 % at one and two years, respectively.

The c-index for the CTP score was 0.66 (95 % CI: 0.52–0.79), while the IGF1-CTP score was slightly higher at 0.68 (95 % CI: 0.55–0.81); however, the comparison of the two c-indexes yielded a slight difference of 0.02 with a corresponding Z-score of 0.33, and the p-value of 0.744, indicating that the difference in predictive performance between the two scoring systems is not statistically significant.

Additionally, no significant correlation was observed between IGF-1 levels and several variables including liver volume (r = 0.22, p = 0.17), tumor volume (r = −0.054, p = 0.75), age (r = 0.17, p = 0.27), BMI (r = 0.13, p = 0.42), INR (r = −0.091, p = 0.56), alanine aminotransferase (ALT) (r = −0.16, p = 0.3) or AFP (r = −0.029, p = 0.86). Conversely, IGF-1 levels showed a significant positive correlation with serum albumin (r = 0.41, p = 0.0066) and negative correlations with total bilirubin (r = −0.39, p = 0.011) and aspartate aminotransferase (AST) levels (r = −0.49, p = 0.0011).

**Fig. 1:**
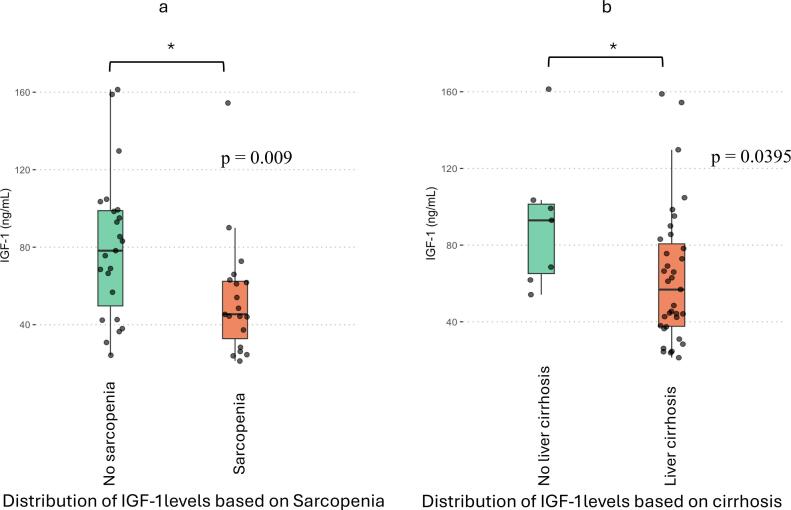
Boxplot graphs showing the IGF-1 distributions between patients without and with sarcopenia (a) and patients without and with cirrhosis (b). The Mann-Whitney *U* test compared the medians, *: p < 0.05.

Patients with sarcopenia had significantly lower median IGF-1 levels (45.4 ng/ml) than those without sarcopenia (78.2 ng/ml), with a p-value of 0.009 (Fig. 1a).

Furthermore, the median IGF-1 level was significantly higher in patients without cirrhosis (92.5 ng/ml) than those with cirrhosis (56.8 ng/ml), with a p-value of 0.0395 (Fig. 1b). However, there was no significant difference between the median IGF-1 levels for patients with and without prior therapies to SBRT among the cohort, 62,4 and 60 ng/ml, respectively (p-value of 0.88).

### Univariate and multivariate analyses

**Fig. 2:**
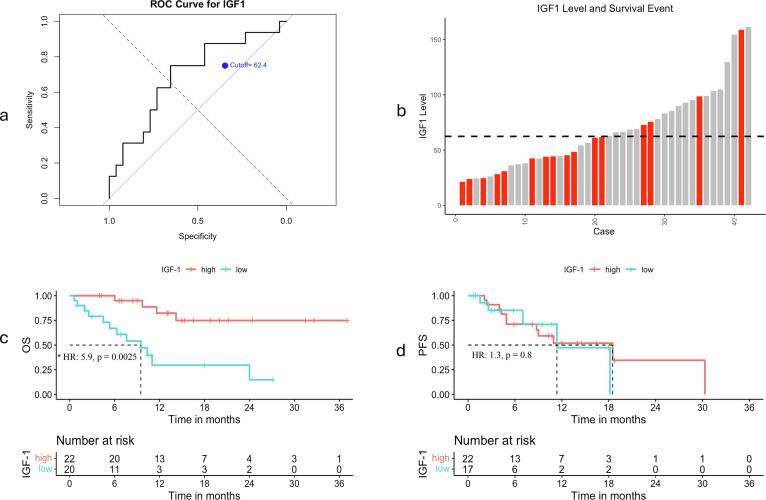
a – ROC curve showing the optional cut-off point of IGF-1 based on survival. b – The histogram represents the distribution of IGF-1 levels across individual cases, with the dashed line indicating the established cutoff point at 62.4 ng/ml. Cases are ordered from lowest to highest expression levels of IGF-1. Red bars signify cases that passed away. c and d: Kaplan Meier curves show the difference for overall survival (OS), and progression-free survival (PFS), respectively, between low and high IGF-1 expression, * p-value < 0.05: statistically significant, Cox-regression. (For interpretation of the references to colour in this figure legend, the reader is referred to the web version of this article.)

Using ROC analysis to optimise the assessment of OS, an IGF-1 cutoff of 62.4 ng/ml was identified for this cohort (Fig. 2). Among patients with IGF-1 levels below this threshold, “the low IGF-1” group, 12 out of 20 patients died, with a significantly higher mortality hazard (HR: 5.9, p = 0.0025) compared to the “high IGF-1” group, only 4 out of 22 patients died (Fig. 2). Despite these differences in OS, PFS did not differ significantly between groups (HR: 1.3, 95 % CI: 0.4–3.4, p = 0.8) (Fig. 2).

Other parameters were evaluated for their impact on OS Table 2. Age had an HR of 0.99 (p = 0.8), and Female sex showed a trend for significantly lower hazard than males (HR: 0.28, P = 0.05). Cirrhosis was associated with an increased hazard (HR: 3.5) but was not statistically significant (p = 0.22). Sarcopenia significantly predicted increased hazard (HR: 4.5, p = 0.006). CTP was a significant predictor, with scores of B and C having HRs of 3.4 (p = 0.036) and 5.7 (p = 0.013) compared to score A, respectively. Tumour size had an HR of 1.7 (p = 0.14), but tumour volume was significantly associated with increased hazard, with each cm^3^ increase correlating with a slight yet significant increase in mortality risk (HR: 1.003, P = 0.008). Liver volume did not show a significant association (HR: 1, P = 0.26). Furthermore, macroscopic vascular invasion significantly predicted increased risk (HR: 2.9, P = 0.041). BMI had an HR of 1.03 with a P value of 0.52. There was no meaningful statical difference between patients with and without prior therapy to SBRT (HR: 0.83, p-value: 0.71).

**Table 2:** Univariate and multivariate analysis. HR: hazards ratio, IGF-1: IGF-1 Insulin-like growth factor 1, CTP score: Child-Turcotte-Pugh score, BMI: Body mass index, AFP: alpha fetoprotein.

Parameter	Univariate analysis		Multivariate cox regression analysis	
	HR	P value	HR	P value
Age	0.99 (0.9–1.05)	0.8		
Sex	0.28 (0.1–3.5)	0.05 *		
Cirrhosis	3.5 (0.47–26.8)	0.22		
IGF-1 (Low vs high)	5.9 (1.9–19.7)	0.0025 *	6.9 (1.4–24.5)	p = 0.014*
Sarcopenia	4.5 (1.5–13.1)	0.006 *		
CTP score A vs • B • C	3.4 (1.1–10.5)5.7 (1.7–22.7)	0.036* 0.013*	2.1 (0.59–7.6)1.53 (0.32–7.3)	0.24 0.59
Tumour size	1.7(0.8–3.4)	0.14		
Tumour volume	1.003 (1.001–1.004)	0.008*	1.004 (1.001–1.006)	0.0022*
Liver volume	1 (0.998–1)	0.26		
Macroscopic vascular invasion	2.9 (1.04–8.14)	0.041*		
BMI	1.03 (0.95–1.1)	0.52		
AFP	1 (0.999–1)	0.138		
Denovo SBRT vs previous therapy	0.83 (0.30––2.3)	0.71		

A multivariate Cox proportional hazards model was then performed using the three most significant liver-related parameters identified in the univariate analysis. An IGF-1 level below 62.4 ng/ml was associated with an HR of 6.9 (p = 0.014), indicating a significantly elevated mortality risk. Although higher CTP scores (B and C) were associated with increased HRs compared to score A (HR = 2.1, p = 0.24 for B and HR = 1.53, p = 0.59 for C), these differences did not reach statistical significance. Moreover, tumour volume was a critical determinant of survival (HR = 1.004, p = 0.0022). The c-index of the model was 0.8 (95 % CI: 0.65:0.85).

We also compared two functional liver reserve models to predict survival. The first was the classical CTP, and the second included CTP and IGF-1 “categorised as low vs high” based on 62.4 ng/ml as the cut off point; the c-index was 0.66 and 0.75, respectively. The comparison of the two c-indices yielded a difference of 0.09 with a Z-score of 1.95 and a p-value of 0.05, suggesting that the performance between the scoring systems in predicting the functional liver reserve is marginally statistically significant in favour of CTP + IGF-1 (low vs. high).

### Development and evaluation of the nomogram

**Fig. 3:**
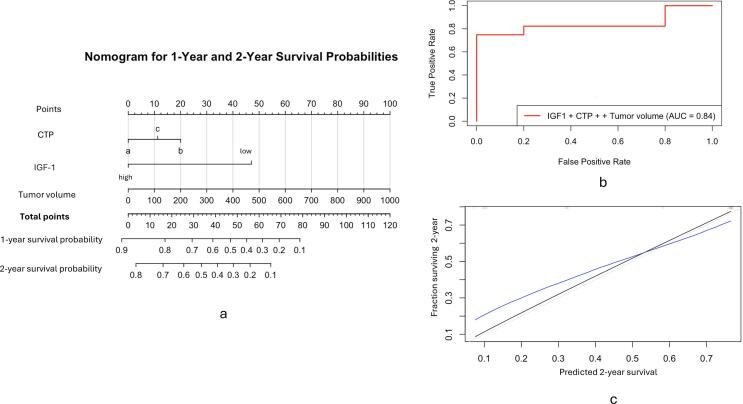
A: Nomogram for predicting the 1- and 2-year survival probability, based on the Child-Turcotte-Pugh score (CTP), IGF-1 category (low vs. high), and Tumor volume in cm^3^. For each patient, the total score was the sum of points for these three factors identified on the points scale. Each patient's 1- and 2-year OS probability was then determined on the total points scale. B: ROC curve respecting the 2-year predictive performance of the model. C: Calibration curve for the nomogram's observed and predicted 2-year survival using the nomogram using 1000 bootstrap resamples. The Black Line represents observed survival, the Blue Line represents the nomogram predictions, and the Gray Line represents ideal Prediction. (For interpretation of the references to colour in this figure legend, the reader is referred to the web version of this article.)

Utilising the multivariate Cox regression model, we developed a nomogram incorporating CTP, IGF-1 (low vs high) and tumour volume to predict patients' 1-year and 2-year survival probabilities. The nomogram assigns point values to each variable based on their prognostic significance, which are summed to derive total points correlating directly with survival probabilities (Fig. 3-a). The model's two-year predictive performance was assessed using time-dependent ROC analysis, which yielded an AUC of 0.84 (Fig. 3-b).

Further, the Calibration of this model at two years involved bootstrap resampling, yielding a mean absolute error of 0.1 with the 0.9 quantiles of absolute errors at 0.064, indicating that 90 % of the predictions deviate by less than 6.4 % from actual outcomes (Fig. 3-c).

## Discussion

The prognostic value of IGF-1 in assessing functional liver reserve in the management of HCC has been investigated in the context of systemic and other local therapies such as thermal ablation or transarterial chemoembolization [21], [22], [23]. However, this study uniquely explores the role of IGF-1 within the specific setting of liver-directed SBRT, marking a novel investigation into its implications in this treatment modality.

It is important to note that although the majority of circulating IGF-1 is bound to IGF-binding proteins (IGFBPs), advancements in immunoassay technology have significantly enhanced the accuracy and reliability of IGF-1 measurements [30]. Historically, the presence of IGFBPs interfered with early assays by hindering antibody binding, but modern techniques—such as the use of IGF-II to displace IGF-1 from binding proteins—have resolved this issue, enabling more precise measurements. Commercial IGF-1 assays are now widely available in clinical practice, offering improved sensitivity and reproducibility [30]. However, variations in assay methods between laboratories still exist, which can influence the interpretation of IGF-1 levels. Therefore, it is recommended to consistently use the same assay method [31].

Unlike other malignancies where higher IGF-1 levels might indicate a higher risk of cancer progression, HCC patients typically have lower serum IGF-1 levels [16], [19]. This trend is attributed to impaired hepatic synthesis resulting from advanced liver disease, which undermines liver function [16], [18], [32], [33]. Consistent with this, our findings indicate IGF-1 was markedly diminished in patients with liver cirrhosis with more severe liver disease and subsequently poorer survival outcomes, aligning with patterns identified in previous research [20].

The study further evaluated the IGF1-CTP classification, which replaces subjective clinical assessments like ascites and encephalopathy with objective IGF-1 serum levels to potentially enhance prognostic accuracy. However, the predictive accuracy of IGF1-CTP was slightly better than the classical CTP score but the difference was not statistically significant. Although this finding aligns with previous reports [22], [24], it still could be attributed to the relatively small number of patients or a difference in the percentage of patients with cirrhosis and its aetiology among the cohort.

Also, IGF-1 levels were markedly lower in those suffering from sarcopenia. This would be attributed to the role of IGF-1, mainly its isoforms IGF-1Ea and IGF-1Eb, which is crucial in promoting muscle hypertrophy and countering age-related muscle deterioration by enhancing autophagy, mitochondrial function, and reducing inflammation [34].

Additionally, IGF-1 levels did not significantly correlate with liver volume, tumour volume, age, BMI, INR, ALT, or AFP, suggesting that IGF-1 does not directly reflect these HCC characteristics or patient status. However, significant correlations were observed between IGF-1 and markers indicative of liver function and damage, such as serum albumin, total bilirubin, and AST, reinforcing the utility of IGF-1 in evaluating liver synthetic capacity [15], [16], [21]. These insights extend the role of IGF-1 beyond mere tumour characteristics, illustrating its broader implications for liver functionality and patient physiological status.

Further, we analysed the optimal cutoff point for IGF-1 based on the survival outcomes of the cohort. A threshold of 62.4 ng/ml was established, dividing the cohort into two groups: a “high IGF-1” group with levels equal to or greater than 62.4 ng/ml and a “low IGF-1” group with levels below this threshold. The OS was significantly higher in the high IGF-1 group than in the low IGF-1 group, with no significant differences in PFS observed between the two groups. These findings underscore the importance of liver reserve assessment in the management of HCC, highlighting liver failure as an essential reason for morbidity and mortality during the management of HCC unrelated to tumour progression [14] and the enhancement in prediction accuracy when combining CTP with IGF-1 categorised as low and high highlights the value of incorporating multiple biomarkers into liver reserve models, which could significantly improve individualised patient management strategies in a clinical setting. While our study identified optimal cut-off at 62.4 ng/ml, different studies suggested different cut-off points for IGF-1, which may reflect the effect of various etiologies of primary liver disease and cirrhosis on IGF-1 levels and may necessitate further validation or individualisation of the optimal cut-off [20], [23], [24], [35].

Furthermore, univariate survival analysis identified male gender, tumour volume, macroscopic vascular invasion, and sarcopenia as factors associated with poorer OS. Subsequent multivariate Cox regression confirmed the statistical significance of IGF-1 (low vs. high) and tumour volume, with the overall model's significant robustness.

Finally, the development of the nomogram, integrating CTP score, IGF-1, and tumour volume, demonstrates substantial predictive power with an AUC of 0.84 for 2-year survival, suggesting a reliable tool for clinical decision-making. The model’s robust calibration, reflected in a mean absolute error of 0.1 and predictions within 6.4 % of actual outcomes for 90 % of cases, underscores its practical utility and accuracy of the nomogram in predicting the survival of the patients.

### Study limitations

Although the study of IGF-1 as a biomarker for the treatment of HCC with SBRT is novel, the main shortcomings of this study were the relatively small sample size and the heterogeneity of the disease groups, which might limit the generalizability of the findings to a broader population. Additionally, despite advancements in IGF-1 assay technologies, there may still be variability in measurement techniques across different assays, which may influence the optimal cut-off point.

## Conclusion

This study underscores the potential of IGF-1 levels as an important biomarker in enhancing the management of HCC by liver-directed SBRT. The findings suggest that lower IGF-1 levels correlate with reduced overall survival, mainly attributed to the limited functional liver capacity without significant differences in disease progression among patients with higher IGF-1 levels. Future directions should investigate possible de-escalation of therapy to those patients to mitigate the limited liver reserve. The analysis reaffirms the importance of a comprehensive clinical evaluation, integrating tumour characteristics and physiological status, to effectively tailor treatment plans.

## Author contributions

Conception and design of the study (AMM, MB, FWRV, MI, MJE), Data collection and analysis (AMM, CS, OB, BPS, KF, PB), Revision of the analysis (MJE, EV, PB), Manuscript drafting (AMM, MW, TV), critical revision of manuscript (TV, OB, MI).

## Declaration of competing interest

The authors declare that they have no known competing financial interests or personal relationships that could have appeared to influence the work reported in this paper.
